# Case Report: Ectopic third molar in the maxillary sinus with infected dentigerous cyst assessed by cone beam CT

**DOI:** 10.12688/f1000research.22466.2

**Published:** 2020-04-16

**Authors:** Khairy Elmorsy, Lubna K. Elsayed, Sara M. El Khateeb

**Affiliations:** 1 Oral and Maxillofacial Surgery Department, Cairo University, Cairo, Egypt; 2 Basic Dental Sciences Department, College of Dentistry, Princess Nourah bint Abdulrahman University, Riyadh, Saudi Arabia; 3 Oral Medicine, Periodontology, Diagnosis and Oral Radiology Department, Faculty of Dentistry, Ain Shams University, Cairo, Egypt

**Keywords:** Ectopic, tooth Eruption, Maxillary Sinus, Molar, Cone Beam CT

## Abstract

Ectopic development of teeth in nondental areas is uncommon, especially in the maxillary sinus. A panoramic radiograph is the routine diagnostic radiographic examination performed for this type of eruption, although cone beam computed tomography (CBCT) is highly recommended for further localization of the ectopic tooth and assessment of the characteristics of any associated lesion before a surgical procedure. We report a case of a 13-year-old female student who presented with purulent discharge posterior to the upper right second molar with a bad taste and foul odour. Radiographic examination revealed a maxillary third molar tooth located at the posterosuperior aspect of the right maxillary sinus with a hyperdense lesion surrounding the crown, obliterating the sinus cavity. Both the tooth and dentigerous cyst were surgically removed under general anaesthesia through Caldwell-Luc antrostomy. After a three-month follow-up, the patient was symptom free and had an uneventful recovery. The rare and critical location of the reported third molar along with the infected dentigerous cyst indicates its complete enucleation to avoid complications as recurrence or malignant transformation.

## Introduction

An ectopic eruption is a condition characterized by the presence of a tooth in a non-dentate area, distant from its usual anatomical location. The aetiology of an ectopic eruption can be unclear; however, several theories have been put forward to describe the rise of this condition, such as: developmental disturbances like cleft palate; pathological processes like large cysts, which displace tooth buds to other areas; odontogenic and rhinogenic infections; or iatrogenic activity ^1– 4^.

The maxillary sinus is an unusual location for an ectopic eruption, although some cases have been reported with unconventional management approaches ^5– 7^.

Ectopic eruption reporting is rare; this case reports the unusual location of the eruption as well as the radiographic assessment and the surgical approach and is a contribution to the literature on this topic.

## Case report

### Initial presentation

**Figure 1.**
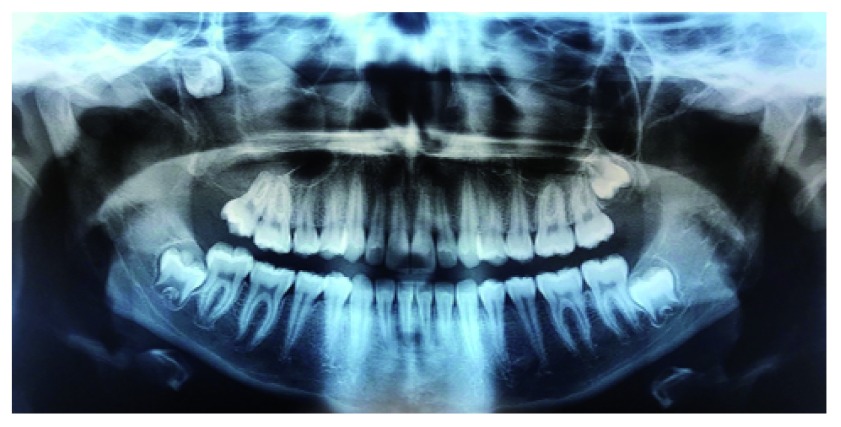
Preoperative panoramic radiograph showing an ectopic eruption of the right maxillary third molar in a superior position near pterygomaxillary fissure inside the right maxillary sinus and surrounded by well-defined hyperdense lesion obliterating the right maxillary sinus cavity.

A 13-year-old Caucasian female student reported to the oral and maxillofacial surgery clinic with the chief complaint of purulent discharge oozing just distal to the upper right second molar, with a bad taste and foul odour, that started two weeks before seeking treatment. Upon clinical examination, the patient had no intraoral or extraoral swelling, and there was a full complement of teeth on that arch except for teeth #18 and #28. All teeth were firm, vital and non-carious. A panoramic radiograph revealed ectopic eruption of the right maxillary third molar in the maxillary sinus with hyperdense lesion surrounding its crown and obliterating the sinus cavity ( Figure 1).

### Diagnostic assessment and intervention

**Figure 2.**
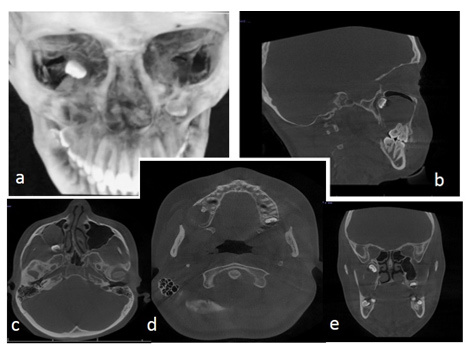
Multiplanar cone beam computed tomography (CBCT) sections: ( **a**) three-dimensional; ( **b**) sagittal; ( **c**, **d**) axial; ( **e**) coronal slices showing the posterosuperior position of the ectopic right third maxillary molar inside the maxillary sinus with associated pericoronal hyperdense lesion and radiographic evidence of oroantral communication due to its destructive effect on the maxillary sinus floor and the alveolar ridge.

Further radiographic investigation using cone beam computed tomography (CBCT) determined the exact location of the maxillary molar and the lesion extension since the molar was seen in close proximity to the infraorbital rim. CBCT showed the ectopic third molar with incompletely formed roots located in the posterosuperior aspect of the right maxillary sinus with a close approximation to the orbital floor superiorly and pterygoid plates posteriorly ( Figure 2a). The third molar was surrounded by a well-defined corticated hyperdense lesion measuring 23×36×35mm, occupying almost the whole cavity of the right maxillary sinus and causing mediolateral expansion of the alveolar ridge. The CBCT also revealed the destructive effect of the associated pericoronal lesion on the right maxillary sinus floor and buccal cortical plate, distal to tooth #17, causing oroantral communication, which explains the reason for the purulent discharge, the chief complaint of the patient ( Figure 2b, 2c, 2d and 2e). Differential diagnosis of the detected lesion was dentigerous cyst, odontogenic keratocyst or Gorlin cyst. Based on the clinical and radiographical examination, the surgical removal of the ectopic third molar along with cyst enucleation was planned through an intraoral approach under general anaesthesia induced via nasopharyngeal intubation. Induction of anaesthesia was achieved with propofol 2mg/kg, fentanyl 1µgm and atracurium 0.5 mg/kg, then maintained with propofol infusion 200µgm/kg/min, fentanyl 1µgm/kg/hour and atracurium 0.1 mg/kg every 20 minutes.

**Figure 3.**
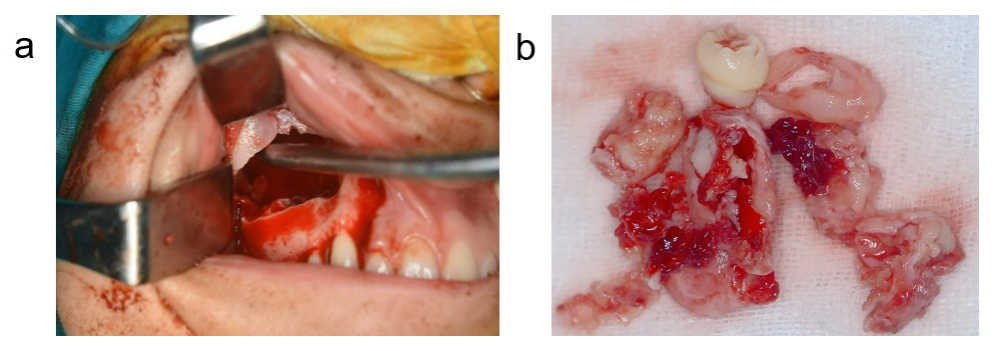
( **a**) Perioperative image showing the bony window created in the anterior wall of the sinus. ( **b**) Underdeveloped ectopic molar with cystic lining.

An oral and maxillofacial surgeon with 15 years of experience performed lateral sinus antrostomy, utilizing a standard Caldwell-Luc approach, with a bony window created in the anterolateral wall of the maxillary sinus ( Figure 3a). An incision was made through the Schneiderian membrane to enter the maxillary sinus. The cystic lining was identified, and the pus was drained prior to the complete removal of the cystic lining and extraction of the ectopic maxillary third molar ( Figure 3b). The antrum was thoroughly irrigated, and the cystic lining was placed in a 10% buffered formalin solution for subsequent histopathological examination and final diagnosis. The flap was closed with chromic catgut sutures with good approximation of edges. Postoperative antibiotics were given to the patient in form of 500 mg of amoxicillin every eight hours for a minimum of five days with an analgesic (325mg acetaminophen every four hours per day), also one dose of betamethasone 4 mg was given to prevent postoperative edema of the cheek, and the patient was instructed not to blow her nose for two weeks.

**Figure 4.**
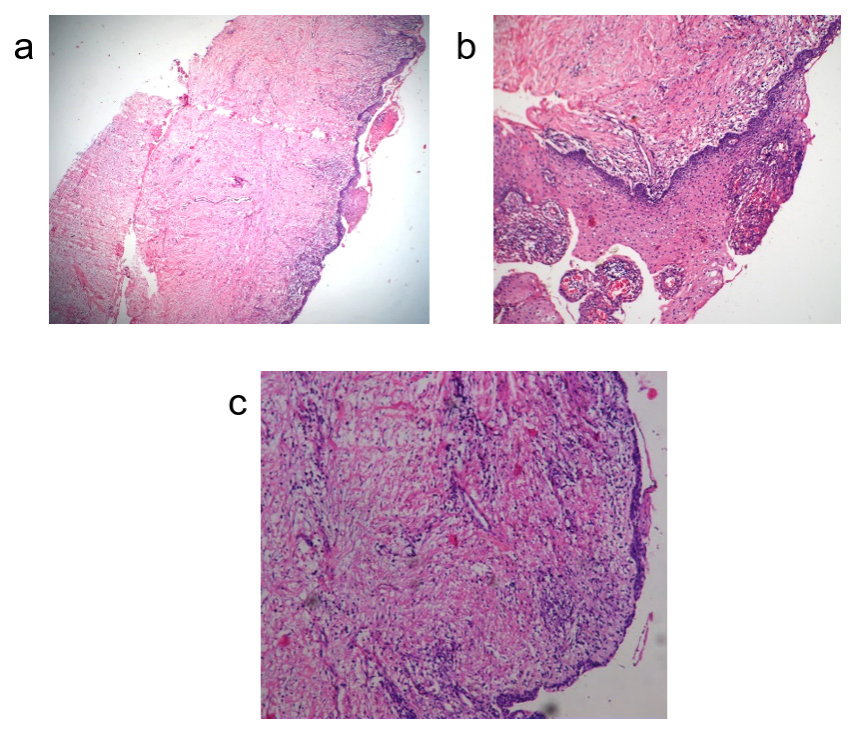
( **a**, **b**) Photomicrograph of infected dentigerous cyst (×4, ×10). ( **c**) Photomicrograph of infected dentigerous cyst showing thin non-keratinized epithelium (×4).

Histopathologic examination reported a cystic cavity lined by a thin non-keratinized stratified squamous epithelium. Part of the epithelial lining showed hyperplasia due to inflammation and long-standing lesion, and a connective tissue wall infiltrated with chronic inflammatory cells and composed of fibroblasts, collagen fibres and blood vessels, suggesting an infected dentigerous cyst ( Figure 4).

### Follow-up

**Figure 5.**
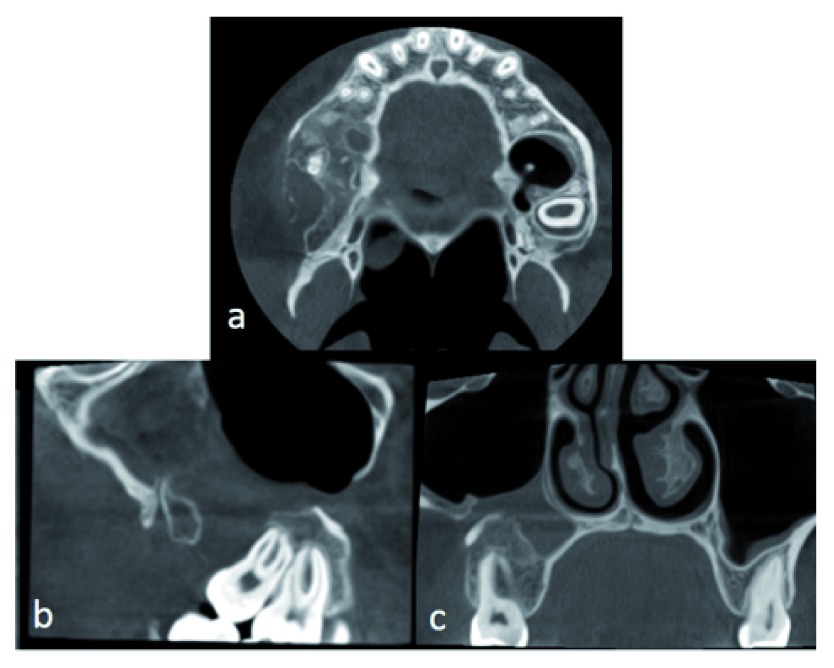
Multiplanar cone beam computed tomography (CBCT) slices: ( **a**) axial cut showing bone formation medially; ( **b**, **c**) sagittal and coronal cuts showing discontinuity of the antral floor and part of the anterior antral wall with oroantral communication starting distal to tooth #16.

A panoramic radiograph and CBCT were performed three months after the surgery. CBCT axial cuts revealed some bone formation in the mediolateral dimension when compared to the preoperative radiographs, which indicates that the bone is in the healing process ( Figure 5a).

Healing appeared to be better clinically than radiographically, the bone requiring longer time to form and be detected radiographically. There is improved bone healing in younger patients, and so we expect a quick healing process with our patient.

Postoperative CBCT scans showed opacification and mucosal lining thickening of the right maxillary sinus, as well as continued discontinuity of the posterolateral floor and part of the anterior wall of the right maxillary sinus ( Figure 5b and 5c).

## Discussion

Ectopic eruption of teeth into dentate regions is relatively common, but such a condition in non-dentate areas like the mandibular condyle, sigmoid notch, or nasal cavity is rare, and the exact aetiology of this phenomenon is not clear ^8^. In our case, we may suggest that the theory of the associated pericoronal pathology with the third molar might have caused its ectopic location.

According to latest review of literature ^2^, 51 patients were reported having ectopic teeth in the maxillary sinus, with the third molars representing the higher prevalence of ectopic teeth, 21 cases. Occasionally, the tooth may erupt into the maxillary sinus and could be asymptomatic ^9^ or cause symptoms such as sinusitis or swelling, facial pain, rhinorrhoea, nasal obstruction and headache ^10^. In the present case, the patient presented with purulent discharge distal to upper second molar and panoramic radiograph showed an ectopic third molar associated with a pericoronal lesion.

Some studies report that panoramic radiography is the radiographic examination of choice in ectopic cases because of its accuracy in detecting these structures and the low level of radiation to which the patient is subjected ^6– 11^. However, the major disadvantage of two-dimensional panoramic radiography is the difficulty in interpreting the exact location of the ectopic tooth and the associated pathology because of the superimposition of different bony structures ^12^.

CBCT presents an accurate three-dimensional imaging modality that offers highly diagnostic images with a sub-millimetre resolution, short scanning time and reduced radiation dose up to 15 times less than multi-slice CT scans ^13^.

The CBCT assessment of our case allowed us to precisely locate the ectopic molar and showed the exact extension and effect of the associated pericoronal lesion on the surrounding structures. This allowed the preoperative identification of the cause of the purulent discharge and aided the surgeon during the surgical procedure, mainly to avoid any surgical complications due to the proximity to the orbital floor and pterygoid plates.

Alqerban *et al.* and Botticelli *et al.* reported better accuracy and significant interobserver agreement in the evaluation of ectopic canine location when using CBCT compared to panoramic radiography ^11, 14^. The smallest field of view matched the clinical indication, recommended to minimize the radiation exposure to the patient ^15^.

In our case, histopathologic examination showed an associated infected dentigerous cyst with the ectopic molar, similar to what has been described previously in the literature, where the dentigerous cysts are the most common pathologic lesion associated with ectopic eruptions ^16^.

Caldwell-Luc antrostomy and full enucleation of the cyst was performed on our patient, as it provides a direct view of the maxillary sinus and allows instrumentation, irrigation and removal of large objects, making it the treatment of choice for surgery at the maxillary sinus ^17^.

Various techniques for managing ectopic teeth are mentioned in the literature, such as endoscopic assisted procedures ^18, 19^, as well as extra and trans-oral approaches ^20^. Endoscopic approaches have less morbidity post and intraoperatively ^21^. Liau *et al.* utilized the endoscopically assisted Caldwell-Luc approach for safer instrumentation of the supero-lateral aspect of the maxillary sinus, allowing direct line access for visualization of potential orbital floor defects. He concluded that this approach allowed accurate visualization of inaccessible areas of the maxillary sinus with the ability to perform surgical intervention under direct line access as required ^19^.

In the present paper, we report a case of infected dentigerous cyst associated with an ectopic molar within the maxillary sinus. Surgical removal for both was performed, and the entire pathologic antral tissue was removed completely and assessed histologically. There were no post-surgical complications, and the patient's postoperative healing was satisfactory.

## Conclusion

Ectopic tooth eruptions in the maxillary sinus are rare. However, its association with dentigerous cysts is relatively common. A standard Caldwell-Luc approach is the management of choice to surgically extract the ectopic tooth along with excision of the pathologic tissue to avoid sinus or ophthalmic complications. Asymptomatic cases should be managed with a similar protocol due to their tendency to form cysts or malignancies.

## Data availability

All data underlying the results are available as part of the article and no additional source data are required.

## Consent

Written informed consent for publication of their clinical details and clinical images was obtained from the parent of the patient.
