# Supplementary material for: Case Report: Ectopic third molar in the maxillary sinus with infected dentigerous cyst assessed by cone beam CT
Source: F1000Res. 2020 Apr 16;9:209. Originally published 2020 Mar 25. [Version 2] doi: 10.12688/f1000research.22466.2 (PMC7135686; doi:10.12688/f1000research.22466.2)
Supplement: Peer review history

# Peer review: Reviewer response for version 1

**DOI:** 10.5256/f1000research.24794.r61700

**Published:** 2020-04-03

**Authors:** Shadia Abdel-Hameed Elsayed

**Affiliations:** 1 Faculty of Dental Medicine for Girls, Al Azhar University, Cairo, Egypt

Thanks for this interesting, fully described, detailed, well written case.The manuscript needs minor revisions.

 Some points need to be declared.

**Title**

 Title need to be summarized and "assessed by cone beam CT" could be removed from the title. CT nowadays used routinely to diagnose the midface lesions for its difficult interpretation on plan panoramic films.

 Case reports are usually describing a very rare condition however dentigerous cyst in the maxillary sinus is not uncommon.

**Figure 3** is an intraoperative photo not perioperative image.

 Usually dentigerous cyst is diagnosed mainly by association with impacted tooth so the conclusion of the paper needs to be more accurate as this case is not ectopic eruption.

 The following paragraph is more accurately describing the present case: In the present paper, we report a case of infected dentigerous cyst associated with an ectopic molar within the maxillary sinus. Surgical removal for both was performed, and the entire pathologic antral tissue was removed completely and assessed histologically. There were no post-surgical complications, and the patient’s postoperative healing was satisfactory.

**Introduction**

 Ectopic eruption definition needs more justification as the present tooth is ectopic but associated with large maxillary cyst and pushed superiorly as result of cyst enlargement.

 Authors did not report any prevalence rate of the dentigerous cyst (which usually associated with impacted wisdom) in the maxillary sinus and also the rate for ectopic impacted teeth in the sinus.

**Operation**

 The cystic lesion had discharge and was diagnosed as infected cyst, does the surgeon treat infection before opening the sinus especially as the cyst was communicate with the oral cavity and possibility of recurrent fistula is high? (Preoperative management of infected sinus is not mentioned).

 Why is nasal anticongestant not prescribed as routine post sinus surgery (note that the sinus membrane appears still edematous in the CBCT)?

Are enough details provided of any physical examination and diagnostic tests, treatment given and outcomes?

Yes

Is the case presented with sufficient detail to be useful for other practitioners?

Yes

Is sufficient discussion included of the importance of the findings and their relevance to future understanding of disease processes, diagnosis or treatment?

Yes

Is the background of the case’s history and progression described in sufficient detail?

Yes

Reviewer Expertise:

Oral and Maxillofacial Surgery

I confirm that I have read this submission and believe that I have an appropriate level of expertise to confirm that it is of an acceptable scientific standard.

# Response

**Published:** 2020-04-04

**Authors:** sara elkhateeb

**Affiliations:** princess Nourah University, Saudi Arabia

**Comment No 1**

Title need to be summarized and "assessed by cone beam CT" could be removed from the title.

**Reply to No 1**

We prefer to keep the title as it is, we think its more enriching to the title to elaborate on the radiographic assessment of the case, especially that we noticed some ectopic cases were not evaluated using CBCT.

**Comment No 2**

Case reports are usually describing a very rare condition however dentigerous cyst in the maxillary sinus is not uncommon.

**Reply to No 2**

Our report is focusing on the ectopic location of the molar “which is a rare condition” and being associated with dentigerous cyst.

**Comment No 3**

Figure 3 is an intraoperative photo not perioperative image.

**Reply to No 3**

The prefix “Peri” means “Intra or during”, so no change will be performed. FYI prefix “Pre” means “before”

**Comment No 4**

Usually dentigerous cyst is diagnosed mainly by association with impacted tooth so the conclusion of the paper needs to be more accurate as this case is not ectopic eruption.

The following paragraph is more accurately describing the present case: In the present paper, we report a case of infected dentigerous cyst associated with an ectopic molar within the maxillary sinus. Surgical removal for both was performed, and the entire pathologic antral tissue was removed completely and assessed histologically. There were no post-surgical complications, and the patient’s postoperative healing was satisfactory.

**Reply to No 4**

a) The term “eruption” means **emission**, **ejection** or **discharge**. Since the molar was clinically “visible” in the maxillary sinus, it is scientifically correct to describe it as “eruption” inside maxillary sinus. Also, the term “ectopic eruption” was previously reported in papers published in indexed journals in same ectopic cases in maxillary sinus.

**Paper examples:**

- Sharma S, Chauhan JS. Bilateral ectopic third molars in maxillary sinus associated with dentigerous cyst: A rare case report. International journal of surgery case reports. 2019 Jan 1; 61:298-301.
- Lombroni LG, Farronato G, Santamaria G, Lombroni DM, Gatti P, Capelli M. Ectopic teeth in the maxillary sinus: A case report and literature review. Indian Journal of Dental Research. 2018 Sep 1;29(5):667.
- Jalal H, Hicham S, Lahcen K, Karim EK. Dentigerous Cyst Associated with an Ectopic Third Molar in the Maxillary Sinus: Report of Cases and Review of Literature. Oral & Maxillofacial Pathology Journal. 2018 Jan 1;9(1).

b) Conclusion preferably to contain a recommendation, your kind suggestion of the paragraph is already included in the paper, so we prefer to keep both.

**Comment No 5 ** 

Ectopic eruption definition needs more justification as the present tooth is ectopic but associated with large maxillary cyst and pushed superiorly as result of cyst enlargement

**Reply to No 5**

We already put general statement in the introduction about the definition of ectopic eruption and the different theories of the causes of its occurrence were discussed in the discussion part. In our case the third molar is in ectopic location and erupted inside the maxillary sinus where the associated pericoronal cyst was the suggested theory for its location.

**Comment No 6**

Authors did not report any prevalence rate of the dentigerous cyst (which usually associated with impacted wisdom) in the maxillary sinus and also the rate for ectopic impacted teeth in the sinus.

**Reply to No 6**

As it is a case report without review of literature, we didn’t go through prevalence of reported conditions, but we agree it is advantageous to be included.

**Comment No 7**

The cystic lesion had discharge and was diagnosed as infected cyst, does the surgeon treat infection before opening the sinus.

**Reply to No 7**

It was a chronic condition due to long standing infection, managing the case in this case will be symptomatic only, so we thought that best managing will be through surgical eradication of the source.

**Possibility of recurrent fistula is high?**

Patient had a 12 months post-surgery follow-up and no fistula was developed.

Why is nasal anticongestant not prescribed as routine post sinus surgery?

**Reply**

It was prescribed.

# Peer review: Reviewer response for version 1

**DOI:** 10.5256/f1000research.24794.r61698

**Published:** 2020-04-02

**Authors:** Najla Dar-odeh

**Affiliations:** 1 Department of Oral Basic and Clinical Sciences, College of Dentistry, Taibah University, Al Madinah Al Munawarah, Saudi Arabia

This study describes a case of a purulent infection that originated in a dentigerous cyst associated with an ectopic third molar in the maxillary sinus. Three diagnostic methods were utilized in this study. A panoramic radiograph was employed initially, followed by a more accurate imaging technique of CBCT to get more detailed information on the extent of the lesion, and to elicit a more accurate differential diagnosis. Finally a histopathological examination was done to achieve a final diagnosis. Successful treatment outcomes were accompanied by a satisfactory follow-up period, and this further increased the chances for a good prognosis.

 The term “ectopic eruption” was used throughout the text, however, taking into consideration the age of the patient and the definition of “eruption”, it is preferable to replace it with the term “ectopic tooth”.

 Literature review was thorough, however, several references were published more than a decade ago. It would be much improved by inclusion of more recent literature, especially case reports that tackle the topic of ectopic third molar in the maxillary sinus, and that take in consideration the young age of the patient.

1. Discussion: first paragraph mentions that exact aetiology is not clear, what do you think the aetiology for ectopic tooth is in your case? 
2. Discussion: How many cases of ectopic teeth in the maxillary sinus were reported so far? And how many of them were for third molars?

I thank authors for the informative manuscript and the solid treatment plan. I also encourage them to write a literature review on this topic.

Are enough details provided of any physical examination and diagnostic tests, treatment given and outcomes?

Yes

Is the case presented with sufficient detail to be useful for other practitioners?

Yes

Is sufficient discussion included of the importance of the findings and their relevance to future understanding of disease processes, diagnosis or treatment?

Yes

Is the background of the case’s history and progression described in sufficient detail?

Yes

Reviewer Expertise:

Oral Medicine.

I confirm that I have read this submission and believe that I have an appropriate level of expertise to confirm that it is of an acceptable scientific standard.

# Response

**Published:** 2020-04-04

**Authors:** sara elkhateeb

**Affiliations:** princess Nourah University, Saudi Arabia

**Comment No 1**

The term “ectopic eruption” was used throughout the text, however, taking into consideration the age of the patient and the definition of “eruption”, it is preferable to replace it with the term “ectopic tooth”.

**Reply to No 1:**

The term “eruption” means **emission**, **ejection** or **discharge**. Since the molar was clinically “visible” in the maxillary sinus, it is scientifically correct to describe it as “eruption” inside maxillary sinus.

Also, the term “ectopic eruption” was previously reported in papers published in indexed journals in similar ectopic cases in maxillary sinus.

**Paper examples:**

-Sharma S, Chauhan JS. Bilateral ectopic third molars in maxillary sinus associated with dentigerous cyst.

—A rare case report. International journal of surgery case reports. 2019 Jan 1; 61:298-301.

-Lombroni LG, Farronato G, Santamaria G, Lombroni DM, Gatti P, Capelli M. Ectopic teeth in the maxillary

sinus: A case report and literature review. Indian Journal of Dental Research. 2018 Sep 1;29(5):667.

-Jalal H, Hicham S, Lahcen K, Karim EK. Dentigerous Cyst Associated with an Ectopic Third Molar in the Maxillary Sinus: Report of Cases and Review of Literature. Oral & Maxillofacial Pathology Journal. 2018 Jan 1;9(1).

**Comment No 2**

Literature review was thorough, however, several references were published more than a decade ago.

**Reply to No 2:**

We agree to some extent with this comment, reference no. 2 will be replaced with:

Lombroni L, Farronato G, Santamaria G, Lombroni D, Gatti P, Capelli M. Ectopic teeth in the maxillary sinus: A case report and literature review. *Indian J Dent Res*. 2018;29(5):667.

For the rest of references, we cannot make any changes in them as we think that having diversity in time ranges in the references and enriches our literature, as we included also 2013/14/18 and 19 articles.

**Comment No 3**

Discussion: first paragraph mentions that exact aetiology is not clear, what do you think the aetiology for ectopic tooth is in your case?

**Reply to No 3**

There is no exact etiology for ectopic teeth development, there are multiple theories to get closer to the exact cause. We will add the following statement to get closer to the etiology of developing ectopic molar in our case:

"In our case, we may suggest that the theory of the associated pathology with the third molar might have caused its ectopic location".

(To be added at the end of the first paragraph in discussion).

**Comment No 4**

Discussion: How many cases of ectopic teeth in the maxillary sinus were reported so far? And how many of them were for third molars?

**Reply to No 4**

According to latest review of literature 2018 (added ref no 2) there were 51 patients were observed, with higher prevalence of ectopic teeth in the third molars, 21 cases.

Lombroni L, Farronato G, Santamaria G, Lombroni D, Gatti P, Capelli M. Ectopic teeth in the maxillary sinus: A case report and literature review. *Indian J Dent Res*. 2018;29(5):667. ****
